# MR-guided LITT therapy in patients with primary irresectable glioblastoma: a prospective, controlled pilot study

**DOI:** 10.1007/s11060-023-04371-x

**Published:** 2023-07-28

**Authors:** Ilaria Viozzi, Christiaan G. Overduin, Anne Rijpma, Maroeska M. Rovers, Mark ter Laan

**Affiliations:** 1 Department of Neurosurgery, Radboud University Medical Center, 6525 GA Nijmegen, The Netherlands; 2 Department of Radiology, Radboud University Medical Center, 6525 GA Nijmegen, The Netherlands; 3 Department of Health Evidence, Radboud University Medical Center, 6525 GA Nijmegen, The Netherlands; 4 Department of Operating Rooms, Radboud University Medical Center, 6525 GA Nijmegen, The Netherlands

**Keywords:** LITT, SLA, Glioblastoma, Pilot, Laser ablation

## Abstract

**Purpose:**

Laser interstitial thermal therapy (LITT) is increasingly being used in the treatment of brain tumors, whereas high-quality evidence of its effectiveness is lacking. This pilot examined the feasibility of conducting a randomized controlled trial (RCT) in patients with irresectable newly diagnosed glioblastoma (nGBM), and generated data on technical feasibility and safety.

**Methods:**

We included patients with irresectable nGBM with KPS ≥ 70 and feasible trajectories to ablate ≥ 70% of the tumor volume. Patients were initially randomized to receive either biopsy combined with LITT or biopsy alone, followed by chemoradiation (CRT). Randomization was stopped after 9 patients as the feasibility endpoint with respect to willingness to be randomized was met. Main endpoints were feasibility of performing an RCT, technical feasibility of LITT and safety. Follow-up was 3 months.

**Results:**

A total of 15 patients were included, of which 10 patients received a biopsy followed by LITT and 5 patients a biopsy. Most patients were able to complete the follow-up procedures (93% clinical, 86% questionnaires, 78% MRI). Patients were planned within 3 weeks after consultation (median 12 days, range 8–16) and no delay was observed in referring patients for CRT (median 37 days, range 28–61). Two CD ≥ 3 complications occurred in the LITT arm and none in the biopsy arm.

**Conclusion:**

An RCT to study the effectiveness of LITT in patients with an irresectable nGBM seems feasible with acceptable initial safety data. The findings from this pilot study helped to further refine the design of a larger full-scale multicenter RCT in the Netherlands.

Protocol and study identifier: The current study is registered at clinicaltrials.gov (EMITT pilot study, NTR: NCT04596930).

**Graphical abstract:**

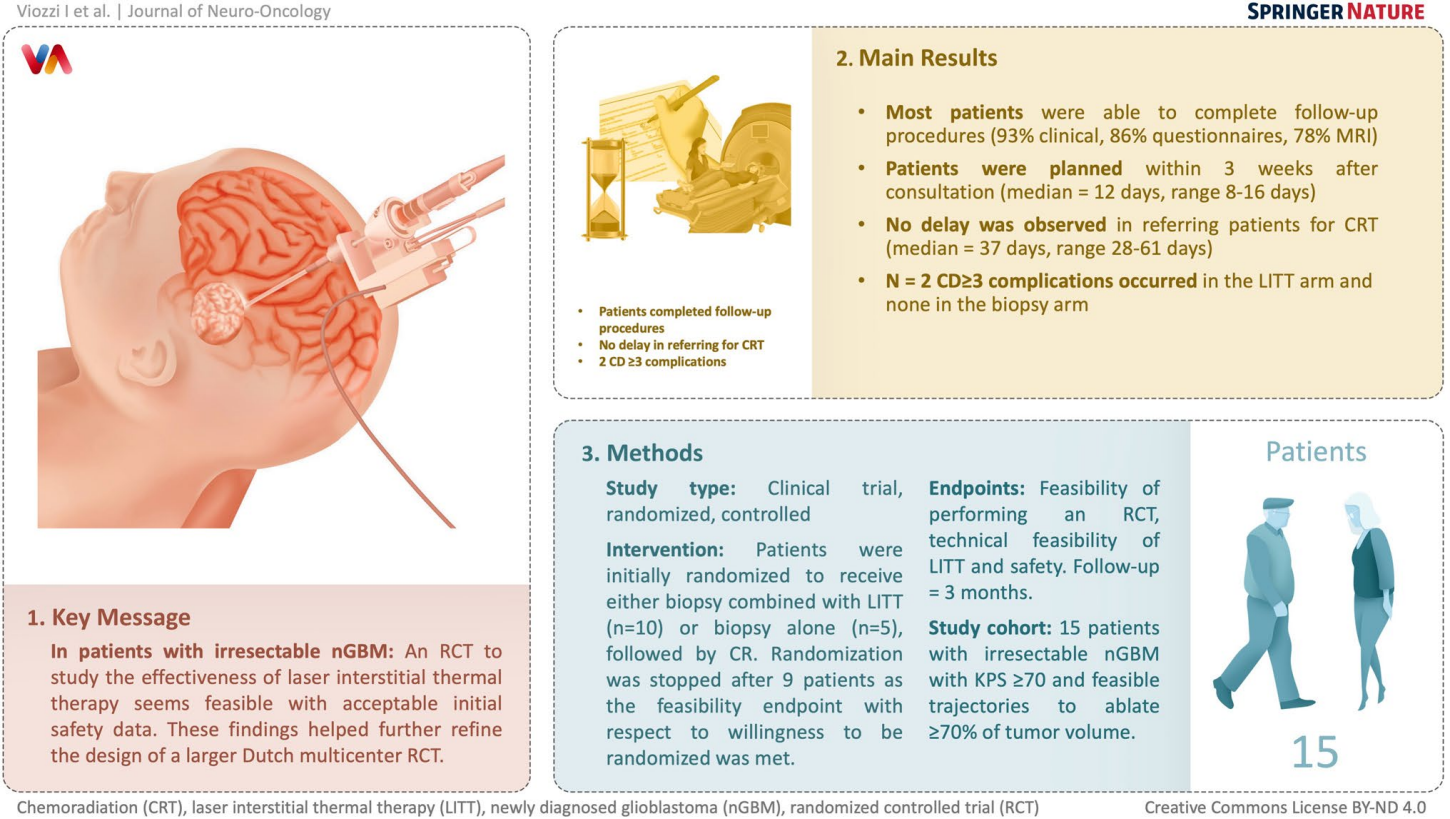

**Supplementary Information:**

The online version contains supplementary material available at 10.1007/s11060-023-04371-x.

## Introduction

Glioblastoma (GBM) is the most common and most aggressive primary malignant brain tumor, with an incidence of 3–5 cases per 100.000 people in Europe [1]. Owing to their aggressive nature, these tumors are responsible for up to 7% of the total life years lost from cancer before the age of 70 [2]. The overall symptom burden and disability for glioma patients are significant, especially in those with high-grade or recurrent disease [3, 4].

Although many efforts have been made to improve current therapies, patients with GBM face a poor prognosis, with a median survival of just 9–18 months [5, 6]. Current standard of care for newly diagnosed glioblastoma (nGBM) patients comprises maximal safe surgical resection followed by adjuvant chemoradiation therapy (CRT) [7]. Maximal resection contributes significantly to overall survival (OS) [6], but data from the Dutch Quality Registry Neurosurgery (QRNS) show that surgery is not deemed feasible in up to 30% of nGBM patients [8]. In these cases, a biopsy is performed to confirm the diagnosis, followed by CRT. According to the current literature, these patients have a significantly worse OS (median 9.2 months) compared to those who undergo subtotal (14.5 months) or gross total resection (18.4 months) before CRT [6, 9].

In recent years, laser interstitial thermal therapy (LITT) has been developed as a minimally invasive treatment for brain tumors, especially in cases where surgery is not deemed safe [10]. Several case series have been published reporting the use of LITT in patients with brain tumors with promising results. Results from systematic reviews and meta-analyses report an OS ranging from 10.2 to 14.2 months in patients with nGBM treated with LITT [11, 12]. However, only small and mostly retrospective cohorts have been published, with high risk of selection and publication bias. No high-quality studies comparing LITT with standard of care are available, precluding any conclusions on cost-effectiveness of LITT [13].

The aim of this pilot study was to examine the feasibility of conducting a randomized controlled trial (RCT) investigating LITT in patients with an irresectable nGBM and to identify any safety or technical feasibility issues to further refine the design of a larger full-scale multicenter RCT.

## Materials and methods

### Study design

The study was initially designed as a prospective randomized pilot study, with randomization between biopsy and LITT in the same session (n = 10) and biopsy alone (n = 10). The main goal of randomization in the pilot study was to determine whether patients with glioblastoma were willing to be randomized for biopsy and LITT versus biopsy only. After including the first 11 patients and treating the first 9 patients, randomization was stopped as the feasibility endpoint with respect to willingness to be randomized was met. From then onwards patients were only included in the LITT arm until the target number of 10 patients in the LITT arm was reached.

This study is reported in accordance with the extension of the Consolidated Standards of Reporting Trials (CONSORT) for randomized pilot and feasibility trials [14].

### Study population

Inclusion criteria were defined as follows:

1. Patients aged ≥ 18 with radiologically suspected diagnosis of supratentorial glioblastoma for whom the multidisciplinary tumor board advised biopsy only and/or patient wished no resection.
2. Maximal volume ≤ 70 cc on post-contrast T1 magnetic resonance imaging (MRI).
3. Safe trajectory/trajectories possible for ablation of at least 70% of the tumor, avoiding eloquent structures or transgression of a ventricle or vessel.
4. Karnofsky Performance Score (KPS) ≥ 70.

Exclusion criteria included:

1. Contra-indication for general anesthesia or MRI.
2. Lesion > 70 cc on post-contrast MRI on the day before intervention.
3. Non-glioblastoma diagnosis as per frozen section analysis or final histology.
4. Pregnancy.

Multifocality and cystic aspect of the tumor were initially defined as exclusion criteria, but the 70% ablation criterium already ensured that highly multifocal and cystic tumors were excluded from the study. Hence these were removed as separate exclusion criteria.

### Study procedures

An extensive description of the study procedures can be found in the supplements. In short, patients were eligible for the study when suspected of a nGBM and the local multidisciplinary tumor board advised biopsy only (e.g., due to deep-seated location like thalamus, basal ganglia, corpus callosum, or eloquent location or multifocal aspect) or patients did not wish a surgical resection. When eligible, patients were informed about the study and, after written informed consent was obtained, formally included and randomized to receive either biopsy alone or biopsy and LITT, both followed by standard adjuvant treatment. After treating the first 9 patients randomization stopped and the remaining patients were planned for LITT. All LITT procedures were performed with the Visualase™ MRI-guided laser ablation system (Medtronic) using the 10-mm diffusing tip LITT probes. Follow-up consisted of scheduled meetings with the treating neurosurgeons 6 weeks and 3 months after surgery. Follow-up MRI was performed at 3 months after surgery. Patients filled in two quality of life questionnaires (QoL) (EQ-5D and EORTC QLQ - BN20) before and 3 months after surgery.

### Outcome measures

Primary outcomes of the study were practical feasibility of a randomized trial in patients with nGBM; safety of the LITT procedure in combination with biopsy; technical feasibility of LITT at our center.

Secondary endpoints of the study were 3-months survival, tumor volume change and change in QoL before and 3 months after treatment.

A detailed description of the chosen outcomes measures is reported in the study protocol (clinicaltrials.gov) and in the supplement of this paper.

### Statistical methods

Descriptive statistics (mean ± standard deviation (SD) or median with an interquartile range for continuous variables, and frequencies or percentages for categorical variables) were computed using SPSS (IBM SPSS Statistics for Windows, Version 25.0. Armonk, NY: IBM Corp) to characterize the sample and examine feasibility outcomes. No comparative analysis was performed due to low sample size.

### Ethical aspects

The study was approved by the Institutional Review Board (METC Oost Nederland). Written informed consent was obtained from all participants before enrolment.

## Results

### Recruitment and feasibility of an RCT

**Fig. 1:**
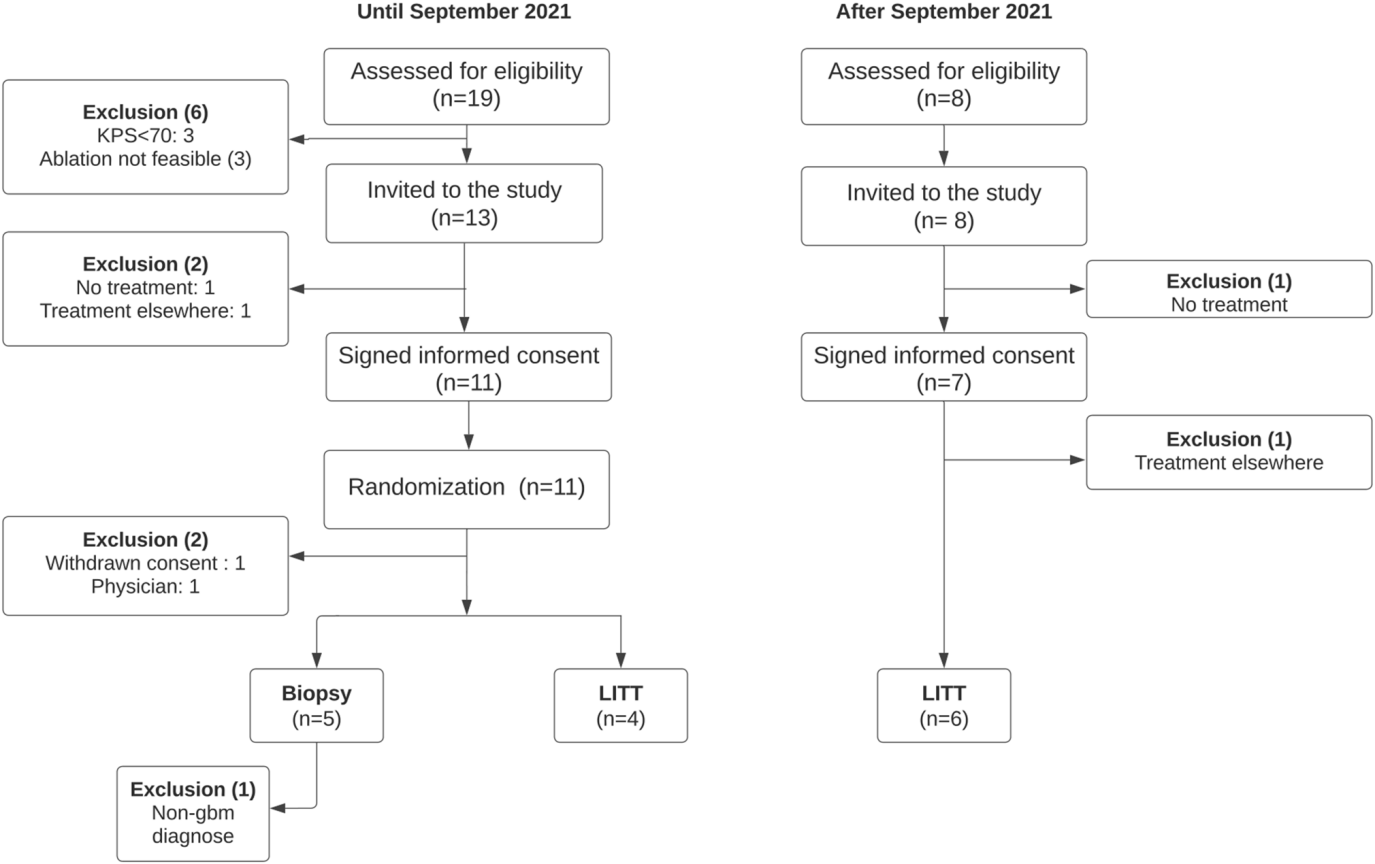
CONSORT Flow-chart showing the inclusion process of the pilot study. *LITT* laser interstitial thermal therapy; *KPS* karnofsky performance score

The trial CONSORT flow-chart of the inclusion process is depicted in Fig. 1.

From November 2020 to February 2022, 27 patients were prospectively screened for eligibility. Of these, 21 met the inclusion criteria (78%), 18 (66%) were included and 15 (55%) were treated.

Until September 2021, 19 patients were screened and 6 did not meet the inclusion criteria (KPS < 70 (n = 3), ablation not feasible (n = 3). Of the 13 patients invited to the study, 11 signed informed consent and were randomized (85%). Two patients decided not to be enrolled; one refused any treatment and one wanted to be treated elsewhere. After randomization 2 patients were excluded, one (randomized in LITT arm) withdrew consent for any treatment and one (randomized in the biopsy arm) developed a psychosis and was considered not mentally competent to continue in the study. With these findings, we considered the randomization feasible in this population and randomization was stopped to maximize experience with LITT. After September 2021, 8 additional patients were assessed for eligibility, and all met the inclusion criteria. One patient refused any treatment and one patient wanted to be treated elsewhere. In total, 15 patients were treated in the study, of which 5 in the biopsy group and 10 in the LITT group. As one patient in the biopsy group was excluded after treatment because of a non-GBM definitive histological diagnosis, 14 patients were included in the analyses. Baseline characteristics are shown in Table 1.

**Table 1:** Baseline characteristics

	Control (n = 4)	LITT (n = 10)
Age (years median, min-max)	56 (46–80)	61 (50–79)
Gender male (%)	3 (75%)	8 (80%)
Tumor volume (cc median, min-max)	2.3 (0.64–17.60)	9.6 (4.50–35)
Tumor location (n)	Frontal (2), Temporal (1), Parietal (1)	Frontal (4), Parietal (5), Occipital (1), Thalamus (1), Corpus Callosum (4)
Tumor lateralization (n)	Left (4)	Right (4), Left (7)
Dexamethasone (mg median, min-max)	2 (0–4)	4 (0–8)
KPS at admission (median, min-max)	90 (80–100)	80 (60–90)

*LITT* laser interstitial thermal therapy; *KPS* karnofsky performance score

The clinical 3-months follow-up was completed by 13 out of 14 patients (93%), 12 patients (86%) filled in the QoL questionnaires, and 11 patients (78%) received a 3-months postoperative MRI.

### Technical feasibility and safety

All patients in the study were treated within three weeks from inclusion and median time from procedure to CRT was 37 days, which in accordance with current national guidelines. Two patients did not start adjuvant treatment, one patient was deceased and the other had a KPS < 70. The median procedure time for biopsy followed by LITT was 206 min. In one case, intra-operative revision was necessary due to malposition. In our series, no disagreement between frozen section and definitive histology was found.

A total of 13 adverse events (AEs) occurred in 8 patients in the LITT arm and in 1 patient in the biopsy arm (see table in supplementary material). Nine AEs were considered not related to the procedure. Four AEs were considered related to the procedure: one patient in the biopsy arm developed a bleeding after biopsy, leading to a mild dysphasia. One patient in the LITT arm developed post-operative edema leading to hydrocephalus (necessitating shunting) and increased neurological deficits (leading to a KPS < 70). One patient in the LITT arm died as a result of progressive bleeding after biopsy. Overall major complication rate (CD ≥ 3) was 20% and one case of post-operative mortality occurred in in the LITT arm. No major complications occurred in the biopsy arm.

An overview of the primary outcomes is depicted in Table 2.

**Table 2:** Summary of primary outcomes of the pilot study

RCT feasibility	
Willingness to be randomized *	11/13 (85%)
Withdrawal within 30 days **	3/18 (17%)
Completed 3-months follow-up***	13/14 (93%)
LITT feasibility	LITT (n=10)
Time inclusion-procedure (days median, min-max)	12 (8-16)
Time procedure-CRT (days median, min-max)	37 (28-61)
Duration procedure (minutes median, min-max)	206 (128-540)
Number of probes (median, min-max)	1.5 (1-3)
Number of intra-operative revision (n)	1
Safety	LITT (n=10)
30-days mortality (n)	1
Complications Clavien Dindo grade ≥ 3 (n)	2
Neurological deficits leading to KPS <70 (n)	1

*Willingness to be randomized has been calculated on the total amount patients who were invited to the study before September 2021

**Withdrawal of consent has been calculated on the total amount of patients who signed the informed consent

***Completed follow-up has been calculated on total patients included in the analysis. Local feasibility and safety have been calculated on the total amount of patients who received LITT.

*RCT* randomized controlled trial; *CRT* chemo-radiotherapy; *LITT* laser interstitial thermal therapy; *KPS* karnofsky performance score

### Secondary outcomes

At 3-months follow-up 13 out of 14 patients were alive and 1 patient in the LITT arm died after a complication. Median KPS worsened in both control and LITT group at 6 weeks and 3-months follow-up.

All patients completed the online QoL questionnaire at screening and 12 out of 14 (86%, 3 in control group, 9 in LITT group) at 3-months follow-up. Patients in both groups showed a worse QLQ-BN20 Mean Total Score and lower EQ-5D Mean Index Value and Mean Visual Analogue Score.

Eleven patients (78%, 3 in the control group and 8 in the LITT group) underwent a 3-month follow-up MRI. Mean tumor volume increased on 3-months post-operative MRI in both groups, both in T2 and T1 with contrast.

An overview of the secondary outcomes is depicted in Table 3. In the supplements a detailed description of the secondary outcomes in individual patients can be found (supplementary Fig. 1).

**Table 3:** Summary of the secondary outcomes of the study. QLQ-BN20 Mean total score: higher scores represent worse quality of life. EQ-5D Mean Index Value: maximum score of 1 indicates the best health state. EQ-5D Visual Analogue Scale: maximum score of 100 indicates the best health state. LITT, laser interstitial thermal therapy; KPS, Karnofsky Performance Score. SD, standard deviation

		Screening		3-months follow-up	
		Biopsy (n = 4)	LITT (n = 10)	Biopsy (n = 4)	LITT (n = 9)
Median KPS (min-max)		90 (80–100)	80 (60–90)	60 (20–80)	70 (30–90)
		Biopsy (n = 4)	LITT (n = 10)	Biopsy (n = 3)	LITT (n = 9)
EORTC-QLQ-BN20 Mean total score (SD)		30.7 (2.3)	38.7 (9.1)	43.3 (16.2)	40.6 (7.6)
EQ-5D Mean Index value (SD)		0.9 (0.1)	0.7 (0.2)	0.5 (0.5)	0.6 (0.2)
EQ-5D Mean vsual analogue score (SD)		67 (23)	83 (12)	45 (34)	49 (18)
		Biopsy (n = 4)	LITT (n = 10)	Biopsy (n = 3)	LITT (n = 8)
Tumor volume T1 ml Mean (SD)		6 (8)	13 (10)	28 (26)	34 (30)
Tumor volume T2 ml Mean (SD)		14 (13)	22 (14)	26 (27)	31 (30)

## Discussion

The main findings of this pilot study are: (1) randomization seems feasible in this patient population and adherence to the study protocol and follow-up procedures was good; (2) combining stereotactic biopsy with LITT in the same session is feasible and does not cause any delay in planning surgery or starting adjuvant therapy; (3) the safety profile of the procedure appears in line with results reported in literature for LITT [12] and craniotomy for tumor resection [15].

The inclusion rate of screened patients was 66%, and most of them (85%) were willing to be randomized, which shows that patients are motivated to participate to the study. None of the patients withdrew after the treatment, which suggests that the follow-up procedures were well tolerated. As inclusions were considerably hindered at the beginning of the trial due to the COVID-19 pandemic, our recruitment rate was lower than anticipated with 18 patients in 15 months instead of 20 patients in 12 months.

Two serious complications (CD ≥ 3) occurred in two LITT patients. One case of post-ablation edema leading to hydrocephalus necessitating shunting and new neurological deficits, and one case of progressive post-operative hemorrhage resulting in death. Post-ablation edema is a known complication in LITT (8.82–35.5%) [10, 12, 16, 17], in particular in larger tumors, and is often reversible[10]. Also post-ablation hemorrhage is a commonly described complication after both LITT [12, 18] and stereotactic biopsy [19, 20]. In our case, a limited hemorrhage was noted after biopsy and LITT was continued. Unfortunately, the hemorrhage progressed after ablation and on post-operative day 1 and ultimately led to the patient’s death on post-operative day 2. Although the hemorrhage originated from the biopsy, it is unclear whether the consecutive ablation procedure contributed to the outcome, for example by increasing tissue fragility of the treated area and facilitating hemorrhage expansion. Both complications were discussed with an independent Data Safety Monitoring Board and were considered serious but in line with results reported from other studies. In several studies, complication rates for LITT are pooled for different patient populations (newly and recurrent glioblastoma, metastases and epilepsy) and vary between 13 and 26% [10, 18, 21-23] and might be underreported [23]. Particularly in patients with nGBM the complication rate is shown to be around 33% [12], which is probably due to the deep-seated location of the tumors and possibly to the precarious neurological condition of these patients. The complication rate in our series is comparable to that described for LITT in previous studies [16, 18, 23] and for craniotomies for tumor resection (around 20%) [15, 24, 25]. We considered this safety profile acceptable. Larger studies should keep carefully reporting complications of LITT in this population and their impact on the ability of conducting adjuvant treatments.

Other reported AEs such as DVT and epilepsy were not considered directly related to the procedure as all of them presented more than 30 days after ablation and are often described in patients with glioblastoma [26, 27].

Median KPS and mean QoL scores seem to worsen and mean tumor volumes seem to increase over 3 months in both groups. This probably reflects the aggressive and progressive nature of the disease. No conclusion on differences between the two groups can be drawn given the number of included patients. Even though a trend toward less growth with stable median KPS and quality of life was noticed, an appropriate powered study is necessary to determine the effect of LITT on disease progression.

This feasibility study helped the design of a full-scale multicenter RCT. Five key lessons emerged from our pilot study.

First, multifocality and cystic degeneration were initially considered as exclusion criteria, in line with previously published series [18, 28]. In our experience, LITT was possible in some cases of multifocality when adequate coverage could be achieved with safe trajectories while tumor volume, shape and possible trajectories were more likely to hinder the procedure. The exclusion criterion “cystic degeneration” is also often used in LITT series because heat spread can be difficult to predict in cystic lesions, but glioblastoma’s rarely present as pure cystic lesions [29] and often show necrotic degeneration which should not be a reason for exclusion.

Second, after the post-operative hemorrhage complication, an amendment to the study protocol was made to interrupt the procedure in case of significant bleeding after biopsy. We are aware that in some centers biopsy and ablation are performed in two different sessions, but in our opinion this approach causes several disadvantages, exposing patients twice to a surgical procedure and general anesthesia, delaying the start of adjuvant therapy and incrementing costs.

Third, we noted that prevention of peroperative CSF leakage is essential to avoid laser misplacement. In one case an intraoperative revision was necessary because of an inadequate laser probe position. The inaccuracy was probably due to a combination of a navigation registration error and perioperative cerebrospinal fluid (CFS) leakage, and we were able to correct the position by navigating on the intraoperative MRI. We therefore advise to use the intraoperative MRI scan in case of replacing the probes.

Fourth, evaluation of real ablation volume on post-operative MRI is challenging in patients with glioblastoma, as ablation zone appears as a peripherally enhancing rim on T1-weighted contrast-enhanced images [30], and is difficult to distinguish from the tumor self.

Fifth, 2 out of 14 patients (14%) were unable to complete the QoL questionnaires since their clinical condition was too poor or because the patient had died. In future studies, it should be considered that data on QoL from the clinically most compromised patients might be incomplete.

The major strength of our study is that, as far as we are aware, this is the first prospectively controlled (feasibility) study for LITT in patients with newly diagnosed glioblastoma. As opposed to previous non controlled series, we provide more reliable insights in general applicability of this technique and practicability of designing a larger clinical trial in this population. The most important limitation of this pilot study is the small sample size collected from a single center limiting any precise comparison between groups or extrapolation to other centers. Furthermore, the recruitment rate was affected by the COVID-19 pandemic, so some uncertainty remains about the inclusion rate in the future.

## Conclusion

In conclusion, our pilot study showed that patients with irresectable nGBM showed high willingness to participate in a randomized study investigating LITT in comparison to standard of care, with good adherence to the study procedures. The study protocol was feasible and initial safety data appear acceptable when compared to craniotomy for tumor resection. To study the effectiveness of LITT in patients with nGBM a larger trial is needed. The outcomes of this pilot study helped to further refine the design of multicenter RCT, which is currently recruiting patients in the Netherlands.

### Supplementary Information

Below is the link to the electronic supplementary material.

Supplementary material 1 (DOCX 214.7 kb)

## Data Availability

The data (deidentified patients data, informed consent forms, clinical study report) that support the findings of this study are available on request from the corresponding author after publication, after providing a methodologically sound proposal. The study protocol is available at clinicaltrials.gov.

## Abbreviations

LITT: Laser interstitial thermal therapy
nGBM: Newly diagnosed glioblastoma
RCT: Randomized controlled Trial
CRT: Chemoradiation therapy
CD classification: Clavien Dindo cassification
SD: Standard deviation
OR: Operation room
OS: Overall survival
EQ-5D: EuroQol- 5 dimension
EORTC QLQ-BN20: European organization for research and treatment of cancer quality of life questionary brain module
KPS: Karnofsky performance score
