# Supplementary material for: MR-guided LITT therapy in patients with primary irresectable glioblastoma: a prospective, controlled pilot study
Source: J Neurooncol. 2023 Jul 28;164(2):405–12. doi: 10.1007/s11060-023-04371-x (PMC10522506; doi:10.1007/s11060-023-04371-x)
Supplement: Supplementary file 1 — Supplementary material 1 (DOCX 214.7 kb) [file 11060_2023_4371_MOESM1_ESM.docx]

**Supplementary Information**

1. Methods
   1. Study procedure

Patients with a suspected diagnosis of nGBM for whom the local multidisciplinary tumor board advised biopsy only or patients who did not wish a surgical resection were potentially eligible for this study. The study team reviewed the clinical and radiological information to assess the eligibility criteria. When eligible, patients were informed about the study and after having obtained written informed consent, were formally included in the study and randomized. Randomization for the first 11 patients was performed with a sealed envelopes system. After treating the first 9 patients randomization stopped and the remaining patients were planned for LITT.

In both arms, patients were admitted the day before the surgical procedure and received a standard neuronavigation MRI. In the LITT arm, the neurosurgeon planned the intraoperative trajectory, the tumor volume and the expected ablation volume using iPlannet software (IPlan 3.0 cranial planning software, Brainlab AG, Munich, Germany). More than one trajectory could be planned to achieve >70% ablation of the tumor volume. To calculate expected ablation volume, along each planned trajectory a 2 cm diameter cylinder was created in the iPlannet software and its cross-section with the total tumor volume was calculated. Trajectories were designed to optimize coverage of the lesion shape while avoiding intervening structure. Patients in the LITT-arm received dexamethasone perioperatively to reduce post-ablation edema, which was tapered postoperatively. All surgeries were performed under general anesthesia. All LITT procedures were performed with the Visualase™ MRI-guided laser ablation system (Medtronic) using the 10-mm diffusing tip LITT probes. Through a short skin incision, a small burr hole and durotomy were performed. Stereotactic biopsy was performed conform routine procedure using the VarioGuide™ (BrainLAB® AG, Feldkirchen, Germany). In the LITT arm, frozen section analysis was performed to confirm diagnosis. Upon confirmation of glioma diagnosis, the cooling catheter and laser fiber were inserted and secured with a skull anchor screw. Patient was transported to the intraoperative MRI suite and the cooling catheters and laser fiber were connected through a waveguide to the control room, where the Visualase™ was installed. MRI was performed using a 3-T clinical MRI system (Magnetom Skyra, Siemens). T1-weighted gradient-echo (MPRAGE) images were acquired to check the probe positions and fused with preoperative planning MRI using iPlannet software to verify correct probe placement. Once proper functioning of the system was confirmed, the procedure started and was closely monitored through the Visualase workstation. For treatment monitoring, MR thermometry images were continuously acquired during laser delivery. At the end of treatment, FLAIR and T1 MPRAGE -sequences after gadolinium enhancement were acquired. The patient was transferred back to the OR where the bone anchor, cooling catheter and laser fiber were removed, and the wound was sutured.

In the control group, a standard stereotactic biopsy was performed using the Varioguide arm.

In both arms, adjuvant therapy consisted of radiotherapy and/or chemotherapy at the discretion of the multidisciplinary tumor board, according to national and international guidelines. Follow-up consisted of scheduled meetings with the treating neurosurgeons 6 weeks and 3 months after surgery. Follow-up MRI was performed at 3 months after surgery. Patients filled in two quality of life questionnaires (EQ-5D and EORTC QLQ - BN20) before and 3 months after surgery.

- 1. Outcomes measures

Practical feasibility of a randomized trial was assessed by willingness to be randomized, inclusion rate, withdrawal within 30 days and completed follow-up at 3 months. Safety was assessed by 30-day mortality, complications with Clavien Dindo grade ≥ 3 and new neurological deficits leading to KPS < 70. LITT was assumed technically feasible when the following pre-defined criteria were met: no delay in surgical procedure (time from inclusion to procedure ≤3 weeks), surgical procedure duration no longer than 4-8 h (comparable to a standard craniotomy for tumor resection), no delay in planning adjuvant therapy (time from LITT to adjuvant therapy ≤ 6weeks). Secondary endpoints of the study were survival at 3 months, tumor volume change and change in quality of life (QoL) before and 3 months after treatment. Tumor evolution was assessed on MRI at screening and 3 months after surgery on T2 or FLAIR and contrast enhanced T1 sequences. Quality of life was assessed using the EQ-5D and the QLQ-BN20 questionnaires ^16,17^.

1. Results

2.2 Description of secondary outcomes (KPS, EQ-5D VAS, tumor volume)

The median difference in KPS from admission to 3 months follow-up was 30 in the biopsy arm and 10 in the LITT arm. The mean EQ-5D VAS worsened from admission to 3-months follow up of 33 points in the biopsy arm and 15 in the LITT arm. Mean percentage tumor volume increased on T1-gadolinium MRI at 3-months follow-up was 617% in biopsy arm and 99% in the LITT arm. In Supplemental Figure 1 data per patient are available.

**
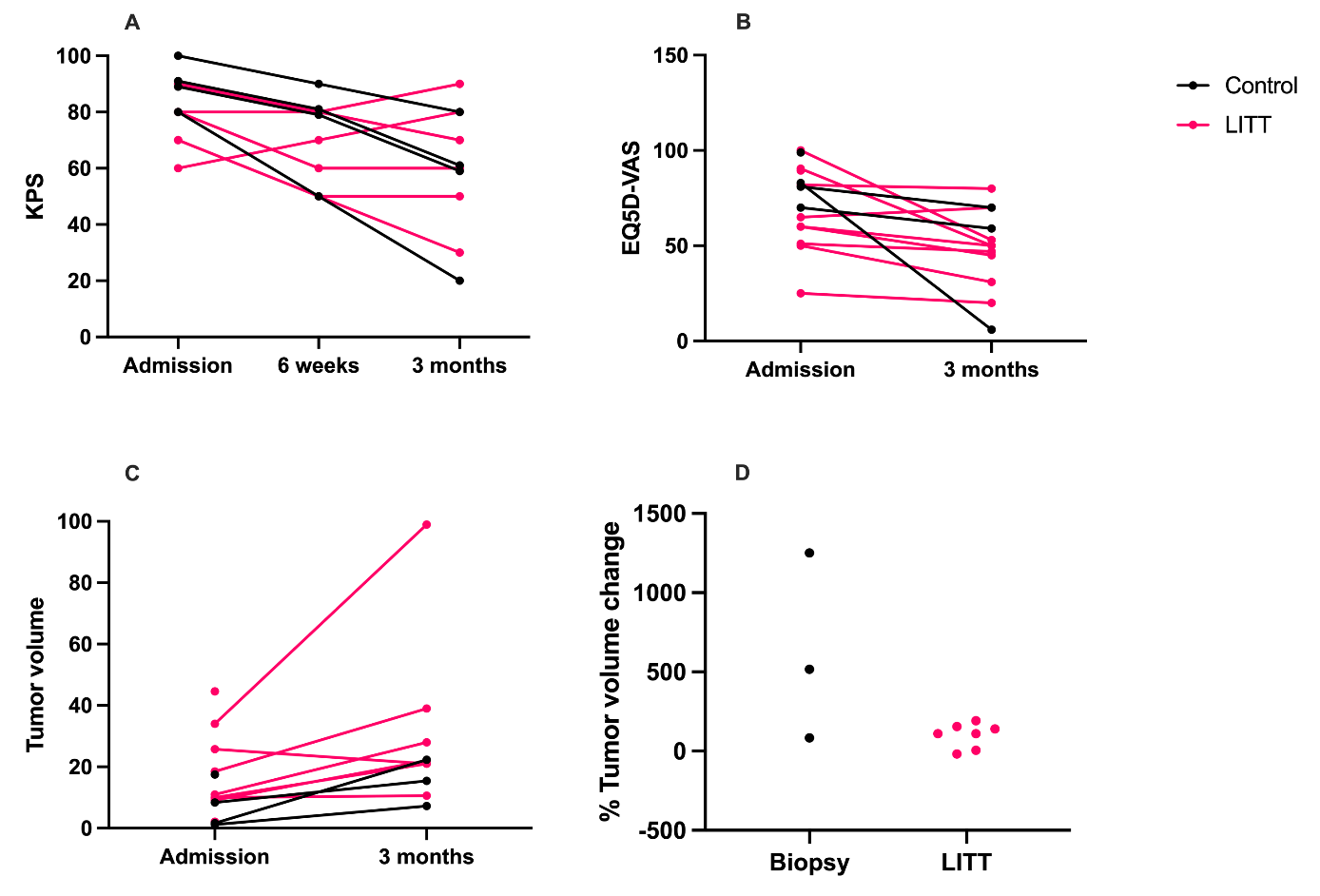
**

Supplementary figure: KPS evolution (A), EQ-5D VAS evolution (B), tumor volume evolution (C) and tumor volume change (D) from admission to 3-months follow-up. Each line or dot represents one patient. In red patients included in the LITT arm, in black patients included in the biopsy arm. LITT, laser interstitial thermal therapy. KPS, Karnofsky Performance Score. EQ-5D-VAS, Visual Analogue Score.

| AE_type | Study arm | AE_relation with procedure | AE_action | AE_outcome | CD |
| --- | --- | --- | --- | --- | --- |
| Neurological deficits | LITT | Definitively related | Only medical intervention | Recovered with major sequelae | 1 |
| Bleeding | Biopsy | Definitively related | None | Recovered with major sequelae | 1 |
| DVT/LE | LITT | Unlikely related | Only medical intervention | Resolved | 2 |
| Epilepsy | LITT | Unlikely related | Renewed hospitalization | Resolved | 2 |
| DVT/LE | LITT | Unlikely related | Renewed hospitalization | Resolved | 2 |
| Epilepsy | LITT | Unlikely related | Renewed hospitalization | Resolved | 2 |
| Epilepsy | LITT | Unlikely related | Renewed hospitalization | Resolved | 2 |
| Epilepsy | LITT | Unlikely related | Renewed hospitalization | Resolved | 2 |
| Epilepsy | LITT | Unlikely related | Only medical intervention | Resolved | 2 |
| Epilepsy | LITT | Unlikely related | Only medical intervention | Resolved | 2 |
| Hydrocephalus | LITT | Definitely related | Surgery | Recovered with major sequelae | 3 |
| DVT/LE | LITT | Unlikely related | Renewed hospitalization | Resolved | 4 |
| Bleeding | LITT | Definitely related | None | Death | 5 |
|  |  |  |  |  |  |

**Supplemental table 1:** Overview of Adverse Events (AE) occurred the study, sorted according to Clavien Dindo (CD) classification. LITT: laser interstitial thermal therapy. DVT/LE: deep venous thrombosis/long embolism
